# A Novel High-Efficiency Natural Biosorbent Material Obtained from Sour Cherry (*Prunus cerasus*) Leaf Biomass for Cationic Dyes Adsorption

**DOI:** 10.3390/ma16124252

**Published:** 2023-06-08

**Authors:** Giannin Mosoarca, Cosmin Vancea, Simona Popa, Mircea Dan, Sorina Boran

**Affiliations:** Faculty of Industrial Chemistry and Environmental Engineering, Politehnica University Timisoara, Bd. V. Parvan No. 6, 300223 Timisoara, Romania; giannin.mosoarca@upt.ro (G.M.); simona.popa@upt.ro (S.P.); sorina.boran@upt.ro (S.B.)

**Keywords:** eco-friendly adsorbent, methylene blue, crystal violet, equilibrium, kinetics, thermodynamic

## Abstract

The present study aimed to investigate the potential of a new lignocellulosic biosorbent material derived from mature leaves of sour cherry (*Prunus cerasus* L.) for removing methylene blue and crystal violet dyes from aqueous solutions. The material was first characterized using several specific techniques (SEM, FTIR, color analysis). Then, the adsorption process mechanism was investigated through studies related to adsorption equilibrium, kinetics, and thermodynamics. A desorption study was also performed. Results showed that the Sips isotherm provided the best fit for the adsorption process of both dyes, with a maximum adsorption capacity of 168.6 (mg g^−1^) for methylene blue and 524.1 (mg g^−1^) for crystal violet, outperforming the capacity of other similar adsorbents. The contact time needed to reach equilibrium was 40 min for both studied dyes. The Elovich equation is the most suitable model for describing the adsorption of methylene blue, while the general order model is better suited for the adsorption of crystal violet dye. Thermodynamic analyses revealed the adsorption process to be spontaneous, favorable, and exothermic, with physical adsorption involved as the primary mechanism. The obtained results suggest that sour cherry leaves powder can be a highly efficient, eco-friendly, and cost-effective adsorbent for removing methylene blue and crystal violet dyes from aqueous solutions.

## 1. Introduction

Water is an essential resource for sustaining life on Earth. Industrial development, urbanization, and population growth have led to an increase in the water requirement. Pollution of water sources, underground and surface, has become a global problem that requires special attention [1,2,3,4].

Among the compounds playing a major role in water pollution are organic substances. Of these, dyes generate significant water pollution [1,3,5,6]. Industries that release considerable amounts of colored wastewater into the environment are textiles, pulp and paper, plastic, leather, cosmetics, pharmaceuticals, rubber, food processing, etc. The dyes have a complex aromatic structure, are stable to light, heat and oxidizing agents, presenting toxic mutagenic, teratogenic and carcinogenic effects on living organisms. Therefore, the elimination of these compounds from wastewater is a necessity [1,5,7,8,9,10,11].

Cationic dyes are more toxic compared to anionic and non-ionic ones due to their ability to interact with negatively charged cell membranes, and present a higher risk for human health [1,5]. Nowadays, methylene blue (MB) and crystal violet (CV) are used in numerous industrial activities, having also important human and veterinary medicine applications. However, their presence in natural waters has a negative impact on aquatic life. They can cause various adverse effects on people, such as irritation of the skin and gastrointestinal system, respiratory problems and high blood pressure, cyanosis, and cancer [1,2,6,9,12].

Many methods (physical, chemical, biological) have been used to remove dyes from aqueous solutions. Adsorption is a popular method widely used in this scope due to its high efficiency, selectivity, and flexibility, as well as its simple design, easy operation, and low cost [13,14,15,16,17,18,19,20,21,22,23,24]. The total cost of the adsorption process is largely determined by the adsorbent material, prompting researchers to seek out inexpensive materials derived from or based on industrial and agricultural waste, minerals, and vegetable materials [13,15,25,26,27,28,29,30].

Numerous plant-based wastes and biomasses have been demonstrated to be effective at retaining dyes. The advantage of these materials compared to other adsorbent categories results from the fact that they are easily-available in large quantities every year in most regions of the globe, are cheap, and do not require additional or preliminary treatment or activation [13,15,18,19,20,22,23,27,31,32].

Cellulose, hemicellulose, pectins, and lignin are the major components of plant leaves. These compounds contain many types of functional groups, including carboxyl, hydroxyl, carbonyl, amino, and nitro able to interact with the dyes functional groups [27].

The sour cherry (*Prunus cerasus*) is a fruit tree belonging to the Rosaceae family that can grow up to a height of 6–10 m. It is native to Europe and Southwest Asia, but it is widely distributed throughout the temperate zones of the globe with well-differentiated seasons. The sour cherry is very well adapted to the winter low temperatures, and the summer drought and high temperatures. Its fruits can be eaten fresh or processed in many forms: juices, jams, compotes, dried fruits, alcoholic beverages, etc. Turkey, the United States, Iran, Italy, Spain, Chile, and Eastern European countries are the main sour cherry growers. Fruits contain significant amounts of sugars, anthocyanin, proteins, mineral salts, vitamins, pectin, and organic acids. They have numerous therapeutic effects, improving the chemical composition of the blood and contributing to delaying the aging process, to curing kidney, diabetic, liver, and cardiovascular diseases. It also has beneficial effects on alleviating mental stress and anemia [33,34,35,36].

Sour cherry leaves are a cost-effective and abundant natural resource that can be found in many areas, making them an excellent option for adsorption that has not yet been reported in the scientific literature. The goal of this study was to show that the adsorbent obtained from this material (without chemical or thermal treatment) can be an effective, economical, and eco-friendly adsorption material for removing methylene blue and crystal violet from aqueous solutions. This was demonstrated by characterizing the materials with FTIR, SEM, and color analysis. Additionally, the effect of various parameters on the adsorption process was analyzed, and studies on equilibrium, kinetics, thermodynamics, and desorption were conducted.

## 2. Materials and Methods

The *Prunus cerasus* L. mature leaves were collected from a sour cherry tree located in a private garden in Cerneteaz village, Timis County, Romania. The leaves were washed with distilled water, dried at room temperature for 5 days, and then placed in an air oven at 90 °C for 24 h. The dried leaves were then ground into a fine powder material with an electric mill, passed through a 2 mm sieve, and washed again with distilled water to remove any turbidity and color. The washed powder material was then dried in an air oven at 105 °C for 8 h.

A Shimadzu Prestige-21 FTIR spectrophotometer (Shimadzu, Kyoto, Japan), a Quanta FEG 250 microscope (FEI, Eindhoven, The Netherlands), and a Cary-Varian 300 Bio UV-VIS colorimeter (Varian Inc., Mulgrave, Australia) were used to carry out FTIR (Fourier-transform infrared spectroscopy), SEM (Scanning Electron Microscopy), and color analysis, respectively. For FTIR analysis, the adsorbent sample was mixed with KBr and formed it into a pellet, while the SEM micrograph was taken at 3000× magnification. The color analysis was conducted under D65 (natural light) illumination and with 10 observer angles. The point of zero charge (pH_PZC_) was identified using the solid addition method [37].

To investigate the adsorption process of each dye, an individual batch system was used. The experiments were carried out in three independent replicates at a constant stirring speed. The pH of the solutions was adjusted with dilute solutions of hydrochloric acid (HCl) and sodium hydroxide (NaOH), both at a concentration of 0.1 (mol dm^−3^), while the effect of ionic strength was tested by adding sodium chloride (NaCl). Finally, the methylene blue and crystal violet concentrations were measured with a UV-VIS spectrophotometer (Specord 200 PLUS UV-VIS spectrophotometer, Analytik Jena, Jena, Germany), at a wavelength of 664 nm and 590 nm, respectively. Limit of Detection (LOD) and Limit of Quantitation (LOQ) for methylene blue concentration determination were 0.21 (mg L^−1^) and 0.61 (mg L^−1^), respectively. For the crystal violet concentration determination, the values for this parameters were LOD = 0.16 (mg L^−1^) and LOQ = 0.49 (mg L^−1^).

Five different isotherm models and five kinetic models were used to analyze the equilibrium and kinetics of adsorption. These models and their equations [38,39] are detailed in the Supplementary Materials, Table S1. The suitability of the tested models was evaluated by determining the value of the determination coefficient (R^2^) and the sum of square error (SSE), chi-square (χ^2^), and average relative error (ARE) [39]. The equations for these error parameters are described in the Supplementary Materials, Table S2. The experimentally obtained results at temperatures of 283, 297, and 317 K were used to calculate the thermodynamic parameters, whose equations [38] are listed in Table S3 of the Supplementary Material.

The desorption process was conducted using three different substances, distilled water, 0.1 (mol dm^−3^), HCl and 0.1 (mol dm^−3^) NaOH, in a batch system with constant stirring for a period of two hours. The desorption efficiency was then calculated using the equation presented in Table S4 in the Supplementary Material.

## 3. Results and Discussion

### 3.1. Adsorbent Material Characteriation

**Figure 1:**
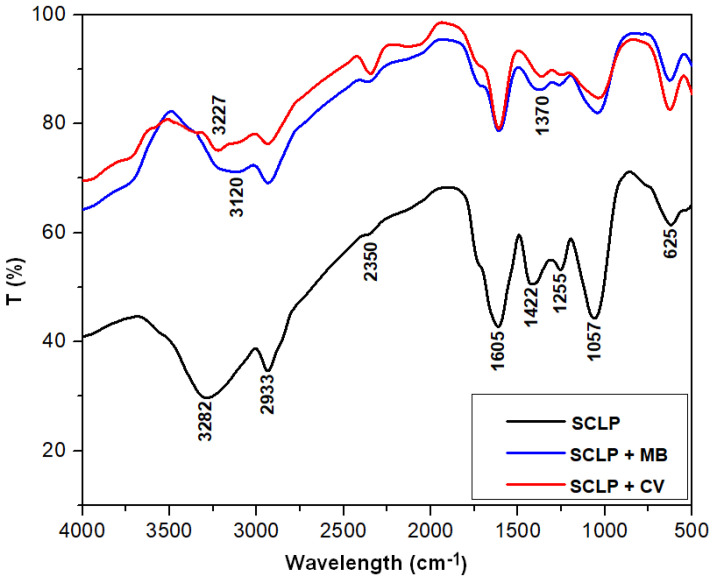
The FTIR spectra of sour cherry (*Prunus cerasus*) leaves powder, before and after dyes adsorption: (SCLP)—sour cherry leaf powder, (SCLP + MB)—sour cherry leaf powder after methylene blue adsorption, (SCLP + CV)—sour cherry leaf powder after crystal violet adsorption.

Figure 1 presents the FTIR spectra of the sour cherry leaves powder (before adsorption). This spectrum shows specific peaks corresponding to different functional groups (Table 1). Analysis of the spectrum indicates that the primary constituents of the adsorbent are cellulose, hemicellulose, and lignin. This fact highlights its affinity to bind dye molecules [27].

After dye adsorption, only two peaks were shifted as follows: 3282 cm^−1^ shifted to 3120 cm^−1^ (methylene blue adsorption) and 3227 cm^−1^ (crystal violet adsorption), respectively; 1422 cm^−1^ shifted to 1370 cm^−1^ at both dye adsorption. These observations suggest that O–H and C–H bonds may be involved in dye retention. The rest of the peaks kept their initial positions and no new ones appeared, indicating no breaking or formation of new bonds after adsorption; therefore, physical adsorption is the main mechanism involved in the process [50,51,52].

**Figure 2:**
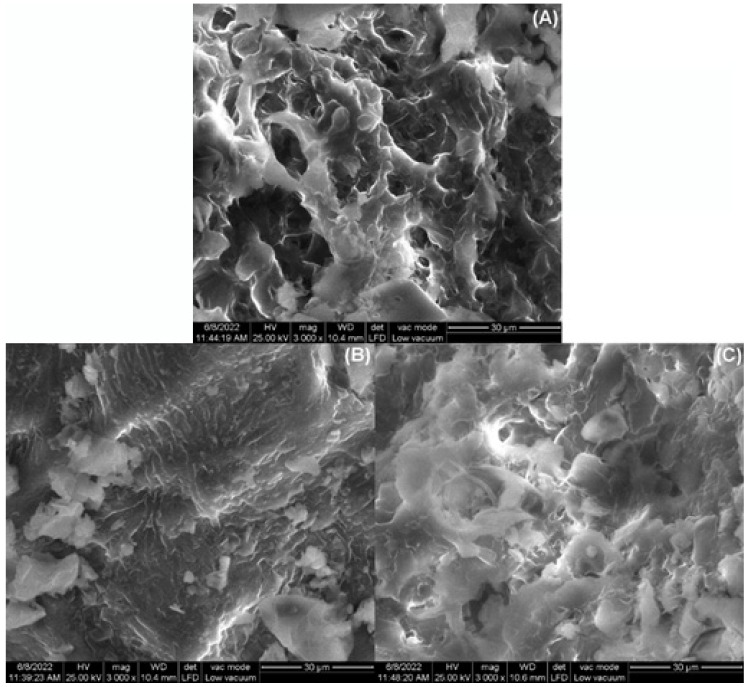
The SEM images of sour cherry (*Prunus cerasus*) leaves powder: (**A**) before adsorption, (**B**) after methylene blue adsorption, (**C**) after crystal violet adsorption (cathode voltage 25 kV; working distance 10.4 mm, magnification 3000×).

The SEM images of the adsorbent material are displayed in Figure 2. Before adsorption, the adsorbent surface appears to be irregular and complex, with pores, crevices, and empty spaces of various sizes and shapes that suggest it is suitable for capturing dyes. After the adsorption process, the adsorbent surface became more uniform, smoother, and consistent, which indicates that the dye molecules filled up the pores and covered up the surface irregularities (Figure 2B,C).

**Figure 3:**
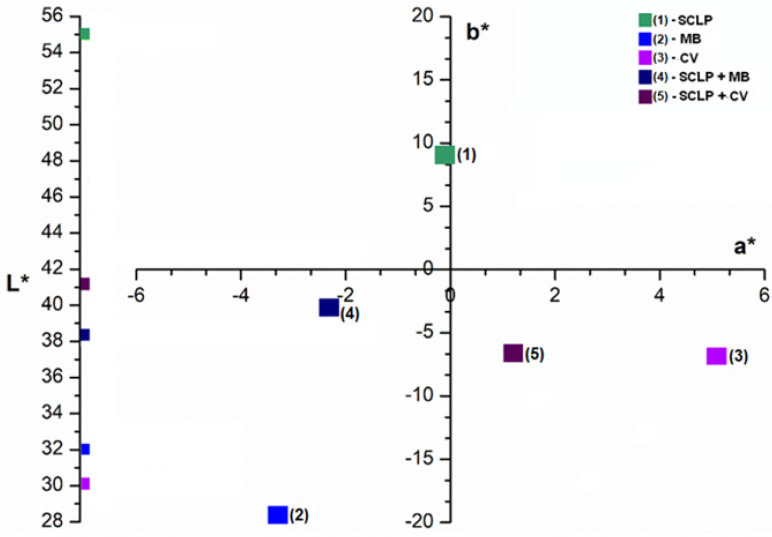
CIEL*a*b* color parameters of sour cherry (*Prunus cerasus*) leaves powder before and after adsorption of methylene blue and crystal violet: 1—(SCLP) sour cherry leaf powder, 2—(MB) methylene blue, 3—(CV) crystal violet, 4—(SCLP + MB) sour cherry leaf powder after methylene blue adsorption, 5—(SCLP + CV) sour cherry leaf powder after crystal violet adsorption).

The adsorption process can be characterized by analyzing the initial and final color of the adsorbent using the CIELab* color parameters. During the adsorption process, the color of the dye in the solution is transferred to the sour cherry leaves powder (Figure 3). This causes the luminosity of the adsorbent to decrease and the color parameters a* and b* to change. Point (1), which describes the initial color of the sour cherry leaves, becomes point (4) after adsorption and shifts into the color area of methylene blue, which was initially represented by point (2). The same observation can be made for the absorption of crystal violet dyes: point (1) becomes point (5) after adsorption and shifts into the color area of crystal violet, which was initially represented by point (3).

**Figure 4:**
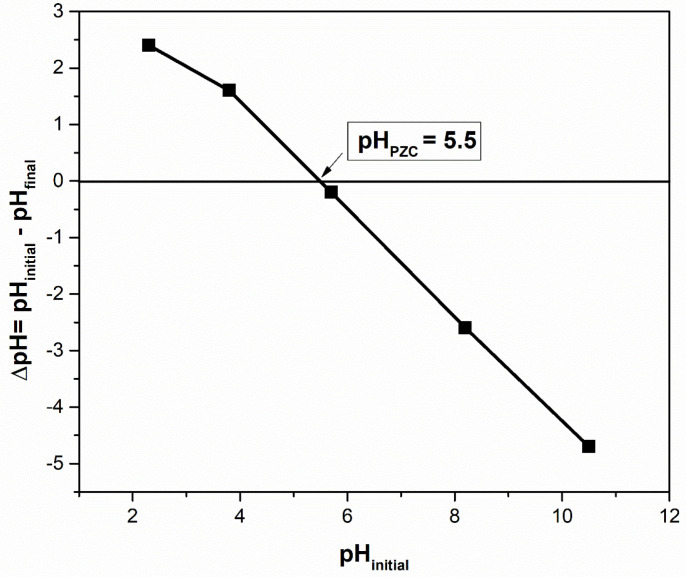
pH_PZC_ determination for sour cherry (*Prunus cerasus*) leaves powder.

The point of zero charge (pH_PZC_) is a measure of the adsorbent surface charge. When the pH is below the pH_PZC_, the surface of the adsorbent becomes positively charged, and when the pH is above the pH_PZC_, the surface becomes negatively charged. The surface charge affects the adsorption of cationic dyes, as a negatively charged surface is more favorable for adsorption [14,23]. According to Figure 4, the pH_PZC_ of the sour cherry leaves powder was determined to be 5.5, meaning that a pH above this value is suitable for the adsorption of methylene blue and violet crystal dyes.

### 3.2. Effect of pH, Ionic Strength, and Adsorbent Dose on Cationic Dyes Adsorption

**Figure 5:**
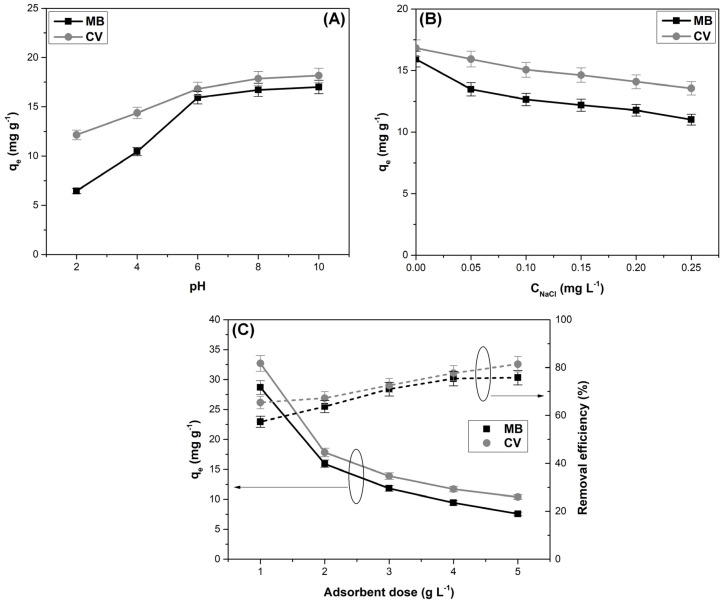
Effect of pH (**A**), ionic strength (**B**), and adsorbent dose (**C**) on methylene blue (MB) and crystal violet (CV) dyes adsorption (the circles group the curves, and the arrows indicate the ordinate to which they relate).

The pH, ionic strength, and adsorbent dose are parameters that significantly influence the dye’s adsorption process. Figure 5 illustrates the effect of these parameters on methylene blue and crystal violet adsorption on sour cherry leaves powder.

As expected, the adsorption capacity was positively influenced when the solutions pH were higher than pH_PZC_, the electrostatic attraction between the cationic dye molecules and the negatively charged adsorbent surface favoring the adsorption process. Similar results were recorded for methylene blue adsorption on pineapple leaf powder [46], citrus limetta peel [13], and lotus leaf powder [53], and for crystal violet dye adsorption on *Ananas comosus* leaves [20], *Ocotea puberula* bark [54], and *Terminalia arjuna* sawdust [14].

The presence of other ions in the dyeing wastewater can have a negative effect on the adsorption process. As illustrated in Figure 5, when the ionic strength is increased, due to the addition of NaCl, the adsorption capacity decreases because the sodium ions are competing with the dye cations for the available adsorption sites on the material surface. A similar effect of ionic strength on the methylene blue and crystal violet adsorption was observed in other studies in which similar adsorbents were used, such as: *Daucus carota* leaves [37], phoenix tree’s leaves [55], potato leaves [56], *Ananas comosus* leaves [46], lotus leaves [53], *Arundo donax* L. [57], and *Artocarpus odoratissimus* leaf-based cellulose [48].

The data in Figure 5 show that higher adsorbent dosages lead to an increase in the adsorption efficiency, based on a larger adsorption surface area and a higher number of active adsorption sites. The decrease in the adsorption capacity is probably due to the fact that many of these sites remain unsaturated and also to the agglomeration of adsorbent material particles [13,55,58,59]. Other researchers previously observed that the amount of adsorbent used had the same effect on the adsorption capacity and removal efficiency of methylene blue and crystal violet [13,14,23,53,54,55].

### 3.3. Equilibrum Study

**Figure 6:**
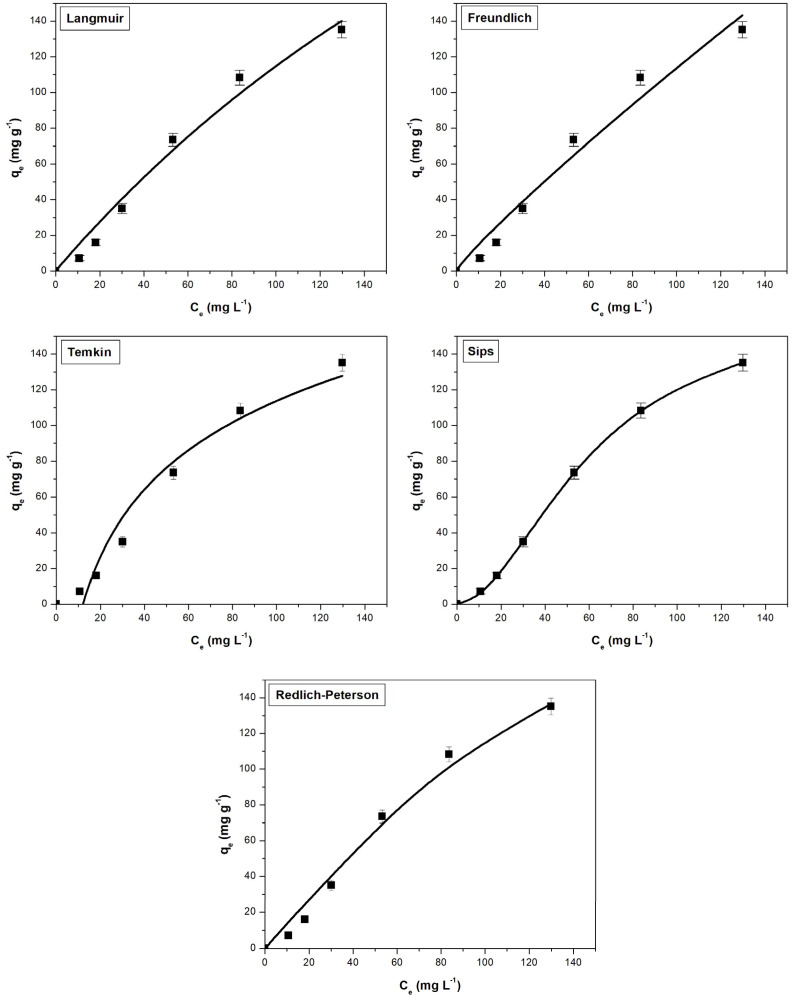
Adsorption isotherms used to assess the adsorption behavior of methylene blue on sour cherry (*Prunus cerasus*) leaves powder.

**Figure 7:**
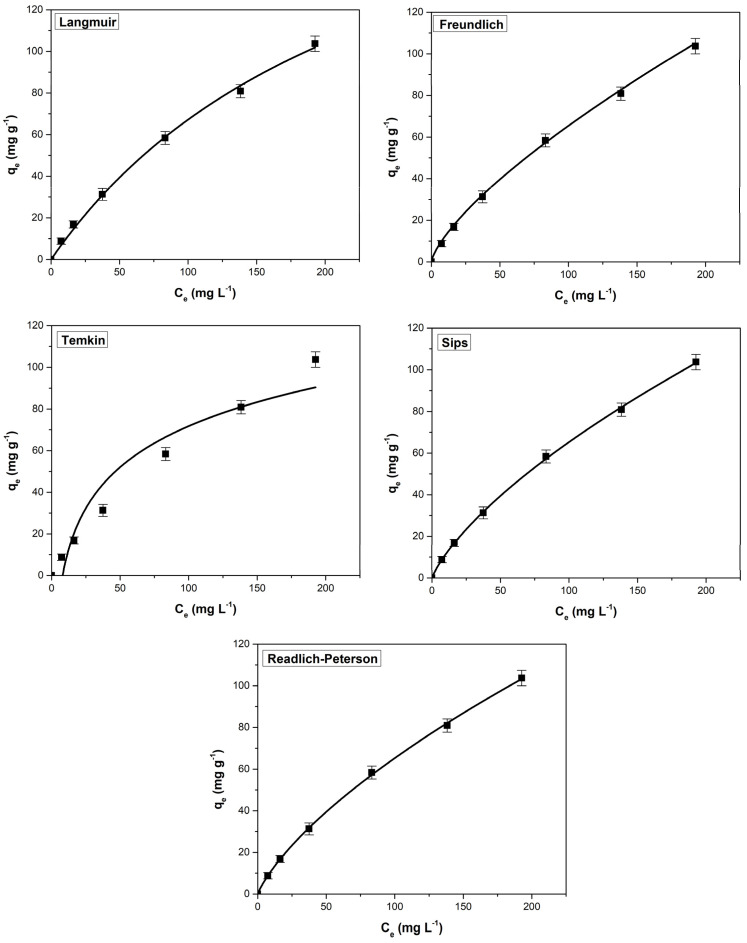
Adsorption isotherms used to assess the adsorption behavior of crystal violet on sour cherry (*Prunus cerasus*) leaves powder.

The equilibrium adsorption process was evaluated using the non-linear isotherms Langmuir, Freundlich, Temkin, Sips, and Redlich–Peterson. After analyzing the fitted isotherm curves (Figure 6 and Figure 7) and the corresponding error parameters (Table 2), it was found that the applicability of the five isotherms for the obtained experimental data follows the order: Sips > Redlich–Peterson > Langmuir > Freundlich > Temkin for the methylene blue adsorption. For crystal violet adsorption, the order of applicability is slightly modified: Sips > Freundlich > Redlich–Peterson > Langmuir > Temkin.

Previous studies showed that the Sips isotherm best characterized the adsorption process of methylene blue on *Maclura pomifera* biomass [60], bilberry leaves [61], raspberry leaves [62], dicarboxymethyl cellulose [63], and the adsorption process of crystal violet dye on *Artocarpus altilis* skin [64], *Eragrostis plana* Nees [65], and motherwort biomass [42].

Table 3 presents a comparison of the maximum absorption capacities of various similar absorbents used for the absorption of methylene blue and crystal violet dyes from aqueous solutions. Analyzing the presented data, it can be seen that the sour cherry leaves powder has a superior adsorption capacity compared to many other similar adsorbents, indicating the practical utility of the new adsorbent proposed in this study.

### 3.4. Kinetic Study

**Figure 8:**
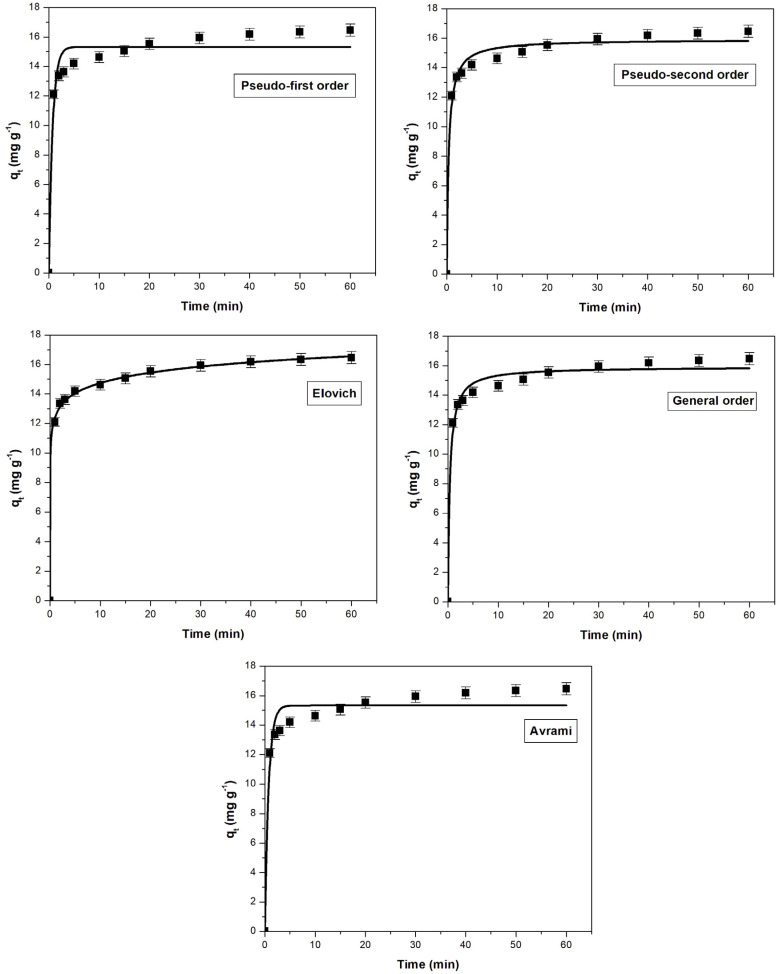
Kinetic models used to assess the adsorption behavior of methylene blue on sour cherry (*Prunus cerasus*) leaves powder.

**Figure 9:**
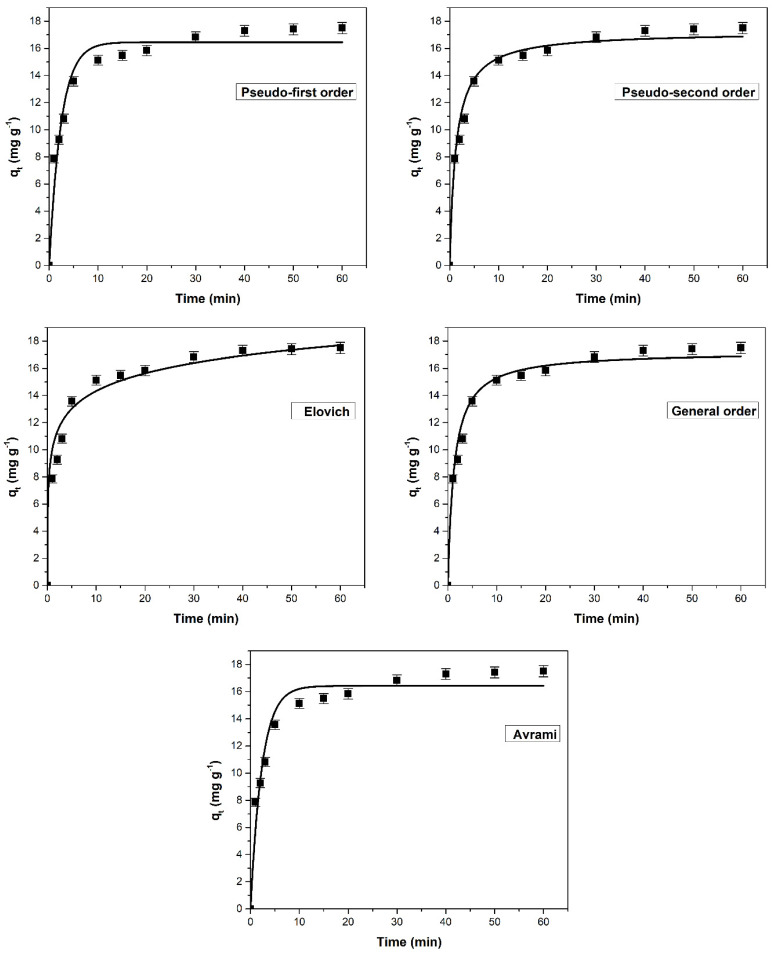
Kinetic models used to assess the adsorption behavior of crystal violet on sour cherry (*Prunus cerasus*) leaves powder.

The effect of contact time on adsorption capacity for methylene blue and crystal violet retention using sour cherry powder as adsorbent material is shown in Figure 8 and Figure 9. During the first 5–10 min of the adsorption process, the capacity of the adsorbent to retain the dyes increased at a rapid rate. As the contact time increased, active adsorption sites gradually filled up, resulting in a slower increase in adsorption capacity. Finally, after 40 min, an equilibrium was reached in which the amount of dye absorbed had stabilized. This suggests that dye diffusion occurred in the pores of the adsorbent and that a monolayer of dye was formed on its surface, resulting in a decrease in the adsorption rate [23,53,86], therefore, the value of the adsorption capacity remained constant.

Table 4 shows comparatively the time taken to reach equilibrium during the adsorption of methylene blue and crystal violet on various similar adsorbents obtained from plant biomass.

The kinetic data for both dyes adsorption were modeled using five different nonlinear kinetic models. Analyzing these models plots (Figure 8 and Figure 9), the constants, and their corresponding error functions (Table 5), it is concluded that the Elovich model is the most appropriate to describe the methylene blue adsorption, while for the adsorption of the crystal violet dye, a more suitable model is the general order. The coefficient of determination (R^2^) values for some tested kinetic models were very similar, however, the lower values for χ^2^, SSE, and ARE are the main arguments that ultimately led to the final conclusion.

### 3.5. Thermodynamic Study

The thermodynamic parameters, calculated from the experimental results obtained at temperatures of 283, 297, and 317 K, are depicted in Table 6. These parameters indicate that the process is spontaneous, favorable, and exothermic, as evidenced by the negative values of the standard Gibbs energy change (ΔG^0^) and the standard enthalpy change (ΔH^0^). Similar results were obtained by other researchers who studied the adsorption of methylene blue on *Salix babylonica leaves* [23], *Daucus carota* leaves [37], potato leaves [56], *Maclura Pomifera* biomass [60], and *Typha angustifolia* (L.) leaves [68] and, respectively, the adsorption of crystal violet on pineapple leaf [21], *Ocotea puberula* bark powder [54], *Arundo donax* L. [57], *Moringa oleifera* pod husk [78], and jackfruit leaf powder [80].

The positive value of the standard entropy change (ΔS^0^) suggests that there is an increased randomness at the solid-liquid interface [13,53,69]. The values of ΔG^0^, both for the adsorption of methyl blue and crystal violet, fall within the range −20 to 0 (kJ mol^–1^). In addition, the ΔH^0^ value is less than 40 (kJ mol^–1^). These two observations indicate that the primary mechanism involved in the absorption is physisorption [23,31,87]. The value of ΔH^0^ lower than 20 (kJ mol^−1^) indicates that van der Waals forces are implied and have an important role in the physical adsorption process [52,88,89].

### 3.6. Desorption Study

**Figure 10:**
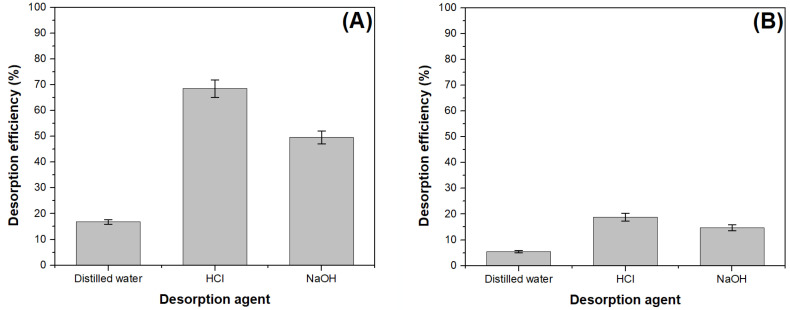
The desorption efficiency for the all tested desorption agents: (**A**) methylene blue loaded adsorbent, (**B**) crystal violet loaded adsorbent.

The data obtained in this study are illustrated in Figure 10. The highest methylene blue desorption efficiency was obtained when HCl was used as desorption agent (Figure 10A). The regenerated adsorbent was reused for methylene blue adsorption, but the obtained adsorption capacity was approximately 50% lower. In conclusion, it can be stated that the regeneration of the adsorbent material is not justified from both technical and economic point of view.

The desorption efficiency of the crystal violet dye was less than 20% regardless of the desorption agent used (Figure 10B). In this case, the regeneration of the exhausted absorbent cannot be considered as feasible.

The fact that sour cherry leaves are a low-cost and readily available material in large quantities in nature compensate for this disadvantage. Furthermore, due to the combustion properties of plant leaves, the incineration of the exhausted adsorbent can be a simple and efficient reuse solution. Another possible use is as a foaming agent to produce ceramic or glass foams. During the combustion process, a large amount of gas results, which makes it an ideal sporogenous precursor for this type of materials [61,62].

## 4. Conclusions

This study proposes a new natural adsorbent material, obtained from mature sour cherry (*Prunus cerasus* L.) leaves, suitable for methylene blue and crystal violet dyes removal from aqueous solutions. This material was characterized and then subjected to adsorption experiments to evaluate its effectiveness in dye removal. The FTIR analysis shows that the adsorbent contains different functional groups specific for cellulose, hemicellulose, and lignin, able to bind dyes. The structure of the adsorbent surface was studied using SEM images, both before and after adsorption, highlighting the importance of the adsorbent porous structure. The dyes retention was indicated using color analysis, dyes color being transferred from the initial solution to the sour cherry leaves powder. pH, ionic strength, and adsorbent dose on cationic dyes adsorption were identified as key factors influencing the effectiveness of the adsorbent. The Sips isotherm best describes the adsorption processes for both studied dyes, with a maximum adsorption capacity of 168.6 (mg g^−1^) for methylene blue adsorption and 524.1 (mg g^−1^) for crystal violet adsorption, superior to other similar adsorbents. The contact time needed to reach equilibrium was 40 min for both studied dyes. The Elovich model is the most appropriate to describe the methylene blue adsorption, while for the adsorption of the crystal violet dye more suitable model is general order. Thermodynamic analyses reveal a spontaneous, favorable, and exothermic process, the calculated values for ΔG^0^ and ΔH^0^ suggesting physisorption as the primary mechanism involved in the absorption process for both dyes.

Regenerating the absorbent is not a viable option, but this fact is compensated by its very low price.

All results indicate sour cherry leaves powder as an affordable, readily available, environmentally friendly, and efficient adsorbent to remove cationic dyes from aqueous solutions.

## Figures and Tables

**Table 1:** FTIR bands that were assigned to different functional groups specific to the main components (cellulose, hemicellulose, and lignin) of the adsorbed material.

FTIR Bands	Assignment	Reference
3282 cm^−1^	stretching vibration of the O–H bonds	[40]
2933 cm^−1^	–CH stretching of CH_2_	[41]
2350 cm^−1^	aromatic ring C=C bond	[42]
1605 cm^−1^	aromatic skeletal and C=O stretch vibrations characteristic of lignin	[43]
1422 cm^−1^	–C–H deformation in lignin	[44,45]
1255 cm^−1^	–C–O stretching and CH or OH bending of hemicellulose structures	[46,47]
1057 cm^−1^	C–O–C stretching of cellulose	[23,48]
625 cm^−1^	bending modes of aromatic compounds	[49]

**Table 2:** Parameters of the adsorption isotherms used to assess the dyes adsorption behavior on sour cherry (*Prunus cerasus*) leaves powder.

Adsorption Isotherm	Parameters	Value	
		MB Adsorption	CV Adsorption
Langmuir non-linear	K_L_ (L mg^−1^)	0.0026 ± 0.0005	0.0041 ± 0.0008
	q_max_ (mg g^−1^)	543.2 ± 16.8	229.8 ± 11.2
	R^2^	0.9846	0.9982
	χ^2^	9.88	1.04
	SSE	317.51	19.96
	ARE (%)	20.72	7.34
Freundlich non-linear	K_f_ (mg g^−1^) (L mg^−1^)^1/n^	1.88 ± 0.27	2.27 ± 0.34
	1/n	0.89 ± 0.08	0.72 ± 0.05
	R^2^	0.9756	0.9994
	χ^2^	11.18	0.18
	SSE	439.62	5.62
	ARE (%)	21.88	2.63
Temkin non-linear	K_T_ (L mg^−1^)	0.31 ± 0.03	0.14 ± 0.02
	b (kJ g^−1^)	94.49 ± 8.34	85.87 ± 6.75
	R^2^	0.8471	0.9411
	χ^2^	69.51	65.19
	SSE	4071.57	600.68
	ARE (%)	41.90	114.97
Sips non-linear	Q_sat_ (mg g^−1^)	168.64 ± 7.41	524.1 ± 16.25
	K_S_ (L mg^−1^)	0.0004 ± 0.0001	0.0033 ± 0.0004
	n	1.86 ± 0.11	0.81 ± 0.08
	R^2^	0.9999	0.9995
	χ^2^	0.09	0.05
	SSE	0.81	3.83
	ARE (%)	2.26	1.04
Redlich-Peterson non-linear	K_RP_ (L g^−1^)	1.34 ± 0.27	1.25 ± 0.24
	a_RP_ (L mg^−1^)	0.0003 ± 0.0001	0.049 ± 0.007
	β_RP_	0.74 ± 0.15	0.62 ± 0.07
	R^2^	0.9928	0.9994
	χ^2^	7.76	0.20
	SSE	191.41	6.12
	ARE (%)	18.24	3.07

*q_m_* and *Q_sat_* are the maximum absorption capacities; *K_L_*, *K_F_*, *K_T_*, *K_S_*, and *K_RP_* are the Langmuir, Freundlich, Temkin, Sips, and Redlich–Peterson isotherms constants; 1/*n_F_* is an empirical constant indicating the intensity of adsorption; *b* is Temkin constant which related to the adsorption heat; *n* is Sips isotherm exponent; *a_RP_* is Redlich–Peterson isotherm constant, *β_RP_* is Redlich–Peterson exponent; *R*^2^ is determining the value of the determination coefficient; *SSE* is the sum of square error; χ^2^ is chi-square and *ARE* is average relative error.

**Table 3:** Comparison of the maximum absorption capacities (q_m_) of various similar absorbents use for methylene blue and crystal violet removal from aqueous solutions.

Methylene Blue Adsorption		
Adsorbent	q_m_ (mg g^−1^)	Reference
raspberry leaves	244.6	[62]
citrus limetta peel	227.3	[13]
lotus leaf	221.7	[53]
bilberry leaves	200.4	[61]
**sour-cherry leaves**	**168.6**	**This study**
*Magnolia grandiflora* leaves	149.2	[66]
banana leaves	109.9	[67]
*Typha angustifolia* leaves	106.7	[68]
*Elaeis guineensis* leaves	103.0	[69]
palm kernel fiber	95.4	[16]
Pará chestnut husk	83.8	[18]
phoenix tree’s leaves	80.9	[55]
*Salix babylonica*	60.97	[23]
*Daucus carota* leaves	66.7	[37]
potato leaves	52.6	[56]
*Ginkgo biloba* leaves	48.1	[70]
rice husk	40.5	[71]
banana stalks	20.8	[72]
*Azadirachta indica* leaves	19.6	[73]
orange peel	18.6	[74]
*Ficcus Palmata* leaves	6.8	[75]
**Crystal Violet Adsorption**		
**Adsorbent**	**q_m_ (mg g^−1^)**	**Reference**
**sour-cherry leaves**	**524.1**	**This study**
water hyacinth root	322.5	[76]
*Ocotea puberula* bark	272.1	[54]
lilac tree leaf	196.7	[77]
bathurst burr biomass	164.1	[52]
*Moringa oleifera* pod husk	156.2	[78]
*Artocarpus altilis* skin	145.8	[64]
motherwort biomass	125.6	[42]
papaya seeds powder	85.9	[79]
Pará chestnut husk	83.6	[18]
palm kernel fiber	78.9	[16]
*Ananasus comosus* leaf	78.2	[20]
*Eragrostis plana* nees	60.1	[65]
jackfruit leaf powder	43.3	[80]
date palm leaves	37.7	[81]
pinus bark powder	32.7	[82]
*Platanus orientalis* leaf	25.8	[83]
*Arundo donax* L.	19.6	[57]
anatolian black pine	12.3	[84]
almond shells	12.2	[31]
*Calotropis procera* leaf	4.14	[85]

**Table 4:** The values of the equilibrium time reported in the scientific literature for the adsorption of methylene blue and crystal violet on various adsorbents obtained from plant biomass.

Methylene Blue Adsorption		
Adsorbent	Equilibrium Time (min)	Reference
potato leaves	24	[56]
*Daucus carota* leaves	30	[37]
orange peel	30	[74]
**sour-cherry leaves**	**40**	**This study**
bilberry leaves	40	[61]
raspberry leaves	40	[62]
Pará chestnut husk	40	[18]
*Magnolia grandiflora* leaves	60	[66]
*Typha angustifolia* leaves	60	[68]
palm kernel fiber	60	[16]
banana stalks	60	[72]
*Azadirachta indica* leaves	60	[73]
*Ficcus Palmata* leaves	80	[75]
*Ginkgo biloba* leaves	100	[70]
*Salix babylonica*	120	[23]
banana leaves	120	[67]
phoenix tree’s leaves	150	[55]
rice husk	150	[70]
citrus limetta peel	180	[13]
lotus leaf	240	[53]
**Crystal Violet Adsorption**		
**Adsorbent**	**Equilibrium Time (min)**	**Reference**
date palm leaves	21	[81]
*Moringa oleifera* pod husk	24	[78]
*Arundo donax* L.	30	[57]
bathurst burr biomass	30	[52]
*Platanus orientalis* leaf	30	[83]
**sour-cherry leaves**	**40**	**This study**
Pará chestnut husk	40	[18]
lilac tree leaf	40	[77]
motherwort biomass	50	[42]
palm kernel fiber	60	[16]
papaya seeds powder	60	[79]
*Calotropis procera* leaf	60	[85]
almond shells	90	[31]
*Ocotea puberula* bark	120	[54]
water hyacinth root	120	[76]
jackfruit leaf powder	120	[80]
pinus bark powder	120	[82]
anatolian black pine	120	[84]
*Eragrostis plana* nees	180	[65]
*Artocarpus altilis* skin	210	[64]

**Table 5:** Parameters of the kinetic models used to assess the dyes adsorption behavior on sour cherry (*Prunus cerasus*) leaves powder.

Kinetic Model	Parameters	Value	
		MB Adsorption	CV Adsorption
Pseudo-first order	k_1_ (min^−1^)	1.33 ± 0.07	0.41 ± 0.05
	q_e,calc_ (mg g^−1^)	15.33 ± 0.78	16.46 ± 0.81
	R^2^	0.9605	0.9636
	χ^2^	0.59	1.35
	SSE	8.77	11.86
	ARE (%)	5.12	7.46
Pseudo-second order	k_2_ (min^−1^)	0.164 ± 0.014	0.034 ± 0.004
	q_e,calc_ (g mg^−1^ min^−1^)	15.90 ± 0.64	17.65 ± 0.95
	R^2^	0.9883	0.9912
	χ^2^	0.17	0.29
	SSE	2.58	2.70
	ARE (%)	2.94	3.74
Elovich	a (g mg^−1^)	0.98 ± 0.17	0.41 ± 0.06
	b (mg g^−1^ min^−1^)	(20.54 ± 0.26) × 10^4^	76.01 ± 5.87
	R^2^	0.9990	0.9828
	χ^2^	0.04	0.40
	SSE	0.23	5.25
	ARE (%)	0.79	4.09
General order	k_N_ (min^−1^ (g mg^−1^)^n–1^)	0.0004 ± 0.0001	0.006 ± 0.001
	q_n_ (mg g^−1^)	16.43 ± 0.35	18.11 ± 0.68
	n	3.89 ± 0.12	2.29 ± 0.18
	R^2^	0.9970	0.9959
	χ^2^	0.05	0.09
	SSE	0.79	1.22
	ARE (%)	1.44	1.99
Avrami	k_AV_ (min^−1^)	0.63 ± 0.05	0.77 ± 0.08
	q_AV_ (mg g^−1^)	15.33 ± 0.34	16.45 ± 0.47
	n_AV_	0.95 ± 0.09	0.53 ± 0.06
	R^2^	0.9605	0.9636
	χ^2^	0.59	1.35
	SSE	8.77	11.86
	ARE (%)	5.11	7.46

*q_t_* is the dye amount adsorbed at time *t*; *k_1_*, *k_2_*, *k_n_*, and *k_AV_* are the rate constants of pseudo-first-order, pseudo-second-order, general order, and Avrami kinetic models; *q_e_*, *q_n_*, and *q_AV_* are the theoretical values for the adsorption capacity; *a* is the desorption constant of Elovich model; *b* is the initial velocity; *n* is the general order exponent and *n_AV_* is a fractional exponent; *R*^2^ is determining the value of the determination coefficient; *SSE* is the sum of square error; χ^2^ is chi-square and *ARE* is average relative error.

**Table 6:** The thermodynamic parameters used to assess the dyes adsorption process.

Dye	ΔG^0^ (kJ mol^−1^)			ΔH^0^ (kJ mol^−1^)	ΔS^0^ (J mol^−1^ K^−1^)
	283 K	297 K	317 K		
MB adsorption	−16.34	−17.05	−17.81	−0.51	5.16
CV adsorption	−17.91	−18.34	−19.55	−0.49	5.83

## Data Availability

All the experimental data obtained are presented, in the form of table and/or figure, in the article and in the Supplementary Materials.

## Supplementary Materials

The following supporting information can be downloaded at: https://www.mdpi.com/article/10.3390/ma16124252/s1, Table S1: The non-linear equations of the adsorption isotherms and kinetic models used to assess the adsorption process, Table S2: The calculation equations for error parameters R^2^, χ^2^, SSE, and ARE, Table S3: The calculation equations of thermodynamic parameters, Table S4: The calculation equation of the desorption efficiency.
