# Supplementary material for: A Novel High-Efficiency Natural Biosorbent Material Obtained from Sour Cherry (Prunus cerasus) Leaf Biomass for Cationic Dyes Adsorption
Source: Materials (Basel). 2023 Jun 8;16(12):4252. doi: 10.3390/ma16124252 (PMC10302337; doi:10.3390/ma16124252)
Supplement: Supplementary file 1 [file materials-16-04252-s001.zip › materials-2413864-supplementary.pdf]

materials

MDPI

Supplementary material

A novel high-efficiency natural biosorbent material obtained from sour cherry (*Prunus cerasus*) leaf biomass for cationic dyes adsorption

Giannin Mosoarca, Cosmin Vancea *, Simona Popa, Mircea Dan * and Sorina Boran

Politehnica University Timisoara, Faculty of Industrial Chemistry and Environmental Engineering, Bd. V. Parvan No. 6, 300223, Timisoara, Romania; giannin.mosoarca@upt.ro (G.M.); simona.popa@upt.ro (S.P.); sorina.boran@upt.ro (S.B.)

* Correspondence: cosmin.vancea@upt.ro (C.V.); mircea.dan@upt.ro (M.D.); Tel.: +40-256404194 (C.V.); +40+40-256404176 (M.D.)

Table S1: The non-linear equations of the adsorption isotherms and kinetic models used to assess the adsorption process.

Isotherm and kinetic models	Equation
Langmuir isotherm	$q_e = \frac{q_m \cdot K_L \cdot C_e}{1 + K_L \cdot C_e}$
Freundlich isotherm	$q_e = K_F \cdot C_e^{1/n_F}$
Temkin isotherm	$q_e = \frac{R \cdot T}{b} \cdot ln(K_T \cdot C_e)$
Sips isotherm	$q_e = \frac{Q_{sat} \cdot K_S \cdot C_e^n}{1 + K_S \cdot C_e^n}$
Redlich-Peterson isotherm	$q_e = \frac{K_{RP} \cdot C_e}{1 + a_{RP} \cdot C_e^{\beta_{RP}}}$
Pseudo-first-order kinetic model	$q_t = q_e(1 - exp^{-k_1 \cdot t})$
Pseudo-second-order kinetic model	$q_t = \frac{k_2 \cdot t \cdot q_e^2}{1 + k_2 \cdot t \cdot q_e}$
Elovich kinetic model	$q_t = \frac{1}{a} ln(1 + a \cdot b \cdot t)$
General order kinetic model	$q_t = q_n - \frac{q_n}{[k_n \cdot (q_n)^{n-1} \cdot t \cdot (n-1) + 1]^{1/1-n}}$
Avrami kinetic model	$q_t = q_{AV}[1 - exp(-k_{AV} \cdot t)^{n_{AV}}]$

where:

- q_m and Q_{sat} are the maximum absorption capacities; K_L, K_F, K_T, K_S and K_{RP} are the Langmuir, Freundlich, Temkin, Sips and Redlich-Peterson isotherms constants; $1/n_F$ is an empirical constant indicating the intensity of adsorption; R is the universal gas constant; T is the absolute temperature; b is Temkin constant which related to the adsorption heat; n is Sips isotherm exponent; a_{RP} is Redlich-Peterson isotherm constant and β_{RP} is Redlich-Peterson exponent which can vary between 0 and 1.

- q_t is the dye amount adsorbed at time t; k_1, k_2, k_n and k_{AV} are the rate constants of pseudo-first-order, pseudo-second-order, general order and Avrami kinetic models; q_e, q_n and q_{AV} are the theoretical values for the adsorption capacity; a is the desorption constant of Elovich model; b is the initial velocity; n is the general order exponent and n_{AV} is a fractional exponent.

Table S2: The calculation equations for error parameters R^2, χ^2, SSE and ARE.

Function name	Equation
Determination coefficient (R^2)	$R^2 = 1 - \frac{\sum_{i=1}^{n}\left(y_{i,exp} - y_{i,mod}\right)^2}{\sum_{i=1}^{n}\left(y_{i,exp} - \overline{y_{i,exp}}\right)^2}$
Sum of square error (SSE)	$SSE = \sum_{i=1}^{n}\left(y_{i,exp} - y_{i,mod}\right)^2$
Chi-square (χ^2)	$\chi^2 = \sum_{i=1}^{n}\frac{\left(y_{i,exp} - y_{i,mod}\right)^2}{y_{i,mod}}$
Average relative error (ARE)	$ARE = \frac{100}{n}\sum_{i=1}^{n}\left\lvert\frac{y_{i,exp} - y_{i,mod}}{y_{i,mod}}\right\rvert$

where: $y_{i,exp}$ is the experimental value; $y_{i,mod}$ is the modeled value; $\overline{y_{i,exp}}$ is the mean values and n is the total amount of information.

Table S3: The calculation equations of thermodynamic parameters.

Thermodynamic parameters	Equation
Standard Gibbs free energy change	$\Delta G^0 = -RT\, ln\, K_L$
Standard enthalpy change<br>Standard entropy change	$ln\, K_L = \frac{\Delta S^0}{R} - \frac{\Delta H^0}{RT}$

where: R is the universal gas constant; K_L is the Langmuir constant and T is the absolute temperature.

Table S4. The calculation equation of the desorption efficiency.

Desorption efficiency equation
$D(\%) = \frac{m_d}{m_a} \cdot 100$

where: m_d is the dye amount liberated by the regenerating agent, m_a is the dye amount adsorbed on the adsorbent material.
